# Differences in Hedonic Responses, Facial Expressions and Self-Reported Emotions of Consumers Using Commercial Yogurts: A Cross-Cultural Study

**DOI:** 10.3390/foods10061237

**Published:** 2021-05-29

**Authors:** Mitali Gupta, Damir D. Torrico, Graham Hepworth, Sally L. Gras, Lydia Ong, Jeremy J. Cottrell, Frank R. Dunshea

**Affiliations:** 1 School of Agriculture and Food, Faculty of Veterinary and Agricultural Sciences, The University of Melbourne, Parkville, VIC 3010, Australia; jcottrell@unimelb.edu.au (J.J.C.); fdunshea@unimelb.edu.au (F.R.D.); 2 Future Food Hallmark Research Initiative Project, The University of Melbourne, Parkville, VIC 3010, Australia; sgras@unimelb.edu.au (S.L.G.); lon@unimelb.edu.au (L.O.); 3 Department of Wine, Food and Molecular Biosciences, Lincoln University, Lincoln 7647, New Zealand; Damir.Torrico@lincoln.ac.nz; 4 Statistical Consulting Centre, The University of Melbourne, Melbourne, VIC 3010, Australia; hepworth@unimelb.edu.au; 5 Department of Chemical Engineering and The Bio21 Molecular Science and Biotechnology Institute, The University of Melbourne, Parkville, VIC 3010, Australia; 6 Faculty of Biological Sciences, The University of Leeds, Leeds LS2 9JT, UK

**Keywords:** biometrics, Cochran’s Q test, ethnic, plant, conscious, unconscious, check-all-that-apply, linear model, correspondence analysis

## Abstract

Hedonic scale testing is a well-accepted methodology for assessing consumer perceptions but is compromised by variation in voluntary responses between cultures. Check-all-that-apply (CATA) methods using emotion terms or emojis and facial expression recognition (FER) are emerging as more powerful tools for consumer sensory testing as they may offer improved assessment of voluntary and involuntary responses, respectively. Therefore, this experiment compared traditional hedonic scale responses for overall liking to (1) CATA emotions, (2) CATA emojis and (3) FER. The experiment measured voluntary and involuntary responses from 62 participants of Asian (53%) versus Western (47%) origin, who consumed six divergent yogurt formulations (Greek, drinkable, soy, coconut, berry, cookies). The hedonic scales could discriminate between yogurt formulations but could not distinguish between responses across the cultural groups. Aversive responses to formulations were the easiest to characterize for all methods; the hedonic scale was the only method that could not characterize differences in cultural preferences, with CATA emojis displaying the highest level of discrimination. In conclusion, CATA methods, particularly the use of emojis, showed improved characterization of cross-cultural preferences of yogurt formulations compared to hedonic scales and FER.

## 1. Introduction

Sensory analysis is important in the food industry, not only for product development but also to guide marketing decisions [1]. While simple sensory testing using a traditional hedonic scale has been widely used to understand the acceptance of foods, this approach has limited freedom for the expression of a full range of sensory experiences [2]. Furthermore, taste responsiveness differs by ethnicity and gender, which may generate different perceptions when tasting the same product. However, these differences may not be reflected when scoring by a hedonic scale and can generate similar scores [3]. Hence, it is important to understand the affective responses of consumers and how people of different cultures perceive food products, especially in a multicultural environment, using a more elaborate approach [4].

A hedonic scale is a simple method of measuring the overall liking scores, which is considered the most informative assessment by a consumer. However, consumer perception of a product goes beyond overall liking, since it also includes extrinsic elements such as the perceived benefits, quality and wellness of the associated food. These extrinsic elements can be explained using more advanced approaches, such as the EsSense Profile^TM^ [5] and check-all-that-apply (CATA)—conscious methods—and the recognition of the facial expression—an unconscious method [6]. The CATA methods can be based on emotion terms or emojis [7,8]. In previous studies, the CATA methodology has been effectively used to compare consumer perception and liking of different chocolate milk desserts and beers and has been shown to be an easy and convenient method for understanding consumer behaviors [9,10]. This method has also been used to observe cultural differences in terms of word associations with the relative quality criteria of rice consumption [11]. An easy, non-verbal CATA method is the use of emoji and emoticons to express emotions towards the different food types [12]. Emoji questionnaires are suitable for a range of populations, as people find emojis to be more expressive compared to emotional terms [7,11]. However, there can be drawbacks with this technique, as it may not detect subtle differences between sensory characteristics [13]. Moreover, this technique is still considered a self-reported conscious method, because it asks consumers to select options from a list and therefore can show bias.

Novel techniques for the assessment of consumer liking involve recording the unconscious facial expressions of consumers during product tasting. This approach can provide details of unconscious emotions expressed by consumers, rather than asking consumers to select emotional terms. Unconscious consumer responses can be measured using facial expression recognition technology, such as FaceReader^TM^ software. This instrumental analysis measures unconscious consumer emotions by reading the face and classifying facial expressions while tasting [14]. It is an easy and quick technique to understand the emotions expressed towards tasted food products. The technique can address any bias, as it records the emotions of the consumers intrinsically [15], although it does not record liking. Facial expression recognition technologies are promising for increasing our understanding of the link between consumer food choices and the facial expressions induced by different the food tastes [16]. In a study, FaceReader^TM^ was used to assess the instant facial expressions of students with an efficacy of 87%. Students were found to experience neutral, sad and angry emotions during assessment [17]. However, this technology can generate noise within the data, which can give false assessments [18].

Benefits may potentially be realized by integrating the different sensory procedures available. Specifically, this may allow a better understanding of consumer perceptions for different products in a multicultural environment. Previous studies have shown a significant effect of culture on the liking and consumer acceptance of a yogurt product and on the form or inclusions preferred by each of the ethnic groups. This makes it significantly important to understand the role or acceptance of the yogurts by different cultures using a cross-cultural sensory experiment [19]. There have been several studies involving various products, such as coffee labels [20], chocolate [21], beer [22] and artificial sweeteners [23], where a combination of implicit and explicit sensory methods have been successfully integrated together to assess the consumer acceptability. In the case of the study with coffee labels, the biometrics technology of facial expression recognition was also shown to be a reliable method for sensory evaluation [20]. However, the sensory analysis of chocolate using other biometrics responses, such as skin temperature and heart rate, did not show any significant differences for the tasted samples [21].

In the present study, dairy yogurts and their plant alternatives were taken as a reference for comparison of the different sensory techniques. Previously, plant-based yogurt alternatives have been shown to be perceived differently by consumers, as compared to dairy yogurts [24], which could produce different emotional experiences. The objective of this study was to understand the effect of culture on the conscious (CATA method using emotions and emojis) and unconscious (facial expression recognition) emotional responses. Further, the study sought to compare these methods with a traditional hedonic scale to understand the method which best represents liking towards yogurts. The study also sought to understand the purchase intent and willingness to pay for these yogurt types. The paper is sub-divided into mainly three sub-sections: (1) measuring liking for the yogurts using each of the four methods (hedonic scales, CATA emotions, CATA emojis and FER) individually and comparing across the Asian and Western cultures, (2) comparing and finding relationships between the methods using a linear mixed model and multi-factor analysis and (3) understanding price perception and purchase intent of the tasted yogurts.

## 2. Materials and Methods

The participants were recruited for the tasting sessions through email invitations and all protocols of this study were approved by the Human Ethics Advisory Group (HEAG) of the Faculty of Veterinary and Agriculture Sciences (FVAS) at the University of Melbourne (Ethics ID 1853507.2). Consumers allergic to lactose, or those with any other allergies, such as wheat or nuts, were excluded from the study. Participants were provided with a gift voucher as an incentive for participation.

### 2.1. Samples

Six yogurt samples that differed in terms of taste and texture were selected, so that it was easier to capture the different consumer responses and understand the method that best relates to consumer liking and expectation. Plant-based yogurts are a new category of products, and recent reports are available mentioning that these are not as acceptable as their dairy counterparts [25]. The present study aimed to compare a traditional dairy product with recently popular plant-based alternatives. Furthermore, comparing different forms (drinkable) and inclusions (berry or cookies) in yogurt also might affect the consumer acceptability. Using a focus group study (*n* = 32), the yogurts were selected for the main tasting experiment, which included the popular options available commercially, and also for the selection of emotion terms in the experiment. The product codes, listed in Table 1, represent the following yogurt types: dairy Greek plain (reference), coconut plain (plant), soy plain (plant), drinkable (sweetened), dairy with crunchies (sweetened) and dairy with berry (sweetened).

### 2.2. Participants

A total of *n* = 62 participants answered the questions for the sensory tasting of the six different yogurt samples. The participants included 47% self-identified Western consumers (29) and 53% Asian consumers (33), 68% females (42) and 32% males (20), with ages ranging from 21 to 58 years. The Western consumers were mainly Australian (14), European (8), North/South American (3) and Latin (4), and the Asian consumers were mainly Chinese (19), Indian/Sri Lankan/Bangladeshi (7), Korean (2), Filipino (1), Persian (1), Vietnamese (2) and Malaysian (1). All participants were students or staff members at the University of Melbourne.

### 2.3. Sensory Evaluation and Data Collection

Participants were asked to taste each of the six yogurt samples (~15 g), which were served in plastic containers at an internal temperature of 10 ± 2 °C, labeled with 3-digit codes and presented in a random order. Participants were seated in individual sensory booths (under white natural LED light); the temperature of the booths was maintained at 22 ± 2 °C. Each booth was equipped with a Samsung 18-inch tablet (Samsung Group, Seoul, South Korea) for recording videos to obtain consumer responses using the Bio-sensory app [26], which did not require any calibration. The armed tablets were placed near to face of the participants, with an in-built camera, and could be adjusted according to the participant’s height. When a participant was seated in the sensory booth, it was ensured that their faces were recorded properly in the set-up, at an approximate distance of 45–50 cm from the participant. The investigators who had expertise in sensory ensured at the beginning of the tasting that the participants’ faces were recorded properly in the set-up.

There was a mandatory break of at least 30 s in between the tasting of any two samples for cleansing of the palette with crackers and water. For each product, participants were asked to evaluate overall liking using a continuous 9-point hedonic scale (1—dislike extremely, 5—neither dislike nor like and 9—like extremely). The self-reported emotional responses of participants were recorded using the CATA methodology, where they were asked to choose the terms from a list of emotions provided (Table S1). These emotion terms were divided into positive, negative and neutral categories and were selected using the perceptual mapping technique [27,28]. A similar procedure was implemented for choosing emojis from another list (Table S2), which were representations of popular face scales from other tasting studies [7]. Preliminary research group discussions also helped in reaching the final list of terms of emotions and emojis, which were fitting for the cross-cultural component of the study.

Facial expressions were recorded during the tasting of each yogurt (Table S3). The videos were cut from the point when participants placed the spoonful of yogurt in their mouth until the time it was swallowed (or consumed), showing their first immediate reaction, which was covered in 5–7 s or even less time in most cases. Further, the data were processed using FaceReader^TM^ 8.0 software (Noldus Information Technology, Wageningen, Netherlands) for recording facial emotions. No calibration was required for the FaceReader^TM^ software as it has pre-built models to detect faces and analyze the expressions. The video analysis was carried out using the “general” settings for all the participants, except for the participants from South East Asia, for whom specific settings were used for analysis. Participants were also asked to assess purchase intent and willingness to pay for each product.

### 2.4. Data Analysis

Overall liking for each sample on the hedonic scale, purchase intent and willingness to pay were analyzed with ANOVA, and Fisher’s LSD was used for paired comparisons, with a significance level of 0.05. The CATA analysis for emotions and emojis was performed with the Cochran’s Q test and correspondence analysis using XLSTAT (Addinsoft, New York, NY, USA: version 2020.1.1). Correspondence analysis was carried out by the software using the variables generated in the Cochran’s Q test. Fisher’s exact test was applied to 2 × 2 cross-tables for comparison of the terms in CATA analysis between the two cultures. ANOVA was carried out on biometric emotions, and the PCA biplots were constructed using XLSTAT (Addinsoft, New York, NY, USA: version 2020.1.1). The linear mixed model for each method was created using REML in GENSTAT (VSN International, Hemel Hempstead, UK; version 16), after selecting the top descriptors using all-subsets regression (*p* < 0.05). Overall liking scores generated from hedonic liking were taken as response variables, and the emotion/emoji/biometric variables were taken as fixed factors. The random factors in the mixed model were “participant codes” and “product codes”. If the fitting of the model did not converge numerically, one or both random factors were omitted to obtain convergence. A multi-factor analysis (MFA) was carried out for a combination of CATA emotions, CATA emojis, FER and overall liking using XLSTAT, with the mean values for each of the factors.

## 3. Results

### 3.1. Overall Liking of Yogurt Products

The mean overall liking scores for yogurt products for both Asian and Western participants were higher for the crunchy yogurt and lower for the berry yogurt (Table 1). The liking for the other four yogurt types was in between these two products, with the drinkable yogurt showing a higher liking by Asian consumers compared to the reference yogurt, and the coconut yogurt showing a lower liking compared to the drinkable and soy yogurts. In contrast, Western consumers rated these three samples (soy, drinkable and reference) similarly, with coconut having a lower liking. The interaction of products and culture was not significant (*p* > 0.05); hence, the differences between yogurt samples could not be observed across the two cultures.

### 3.2. Comparison of Emotion Terms (CATA Emotions Method)

The emotional terms expressed by consumers of Western and Asian backgrounds were compared by the Cochran’s Q test (Table 2). The most selected emotional terms (*p* < 0.05) for Western consumers for each product type were ‘trusted’ and ‘dependable’ for the reference, ‘artificial’ and ‘luxury’ for coconut, ‘cheerful’ and ‘uplifting’ for drinkable, ‘cheerful’ and ‘artificial’ for soy, ‘cheerful’ and ‘uplifting’ for cookies and ‘nasty’ and ‘deceitful’ for berry. The distributions of positive, negative and neutral terms are listed in Table S1. In comparison, for Asian consumers, the top selected emotions (*p* < 0.05) for each of the yogurts were ‘basic’ and ‘common’ for the reference, ‘artificial’ for coconut, ‘cheerful’ and ‘basic’ for drinkable, ‘cheerful’ and ‘artificial’ for soy, ‘cheerful’ and ‘luxury’ for cookies and ‘nasty’ and ‘artificial’ for berry. The cookies product generated positive emotion terms, whereas berry generated negative emotions, by both the cultural groups. The emotion terms ‘neutral’ and ‘guilt-free’ were similarly rated by both cultures for all the yogurt products.

Interestingly, the reference product was related more to positive terms by Western consumers and to neutral terms by Asian consumers. The coconut was linked to more negative emotions by Asian consumers but was intermediate for Western consumers. The term ‘common’ was not significantly different for the six yogurt products in the case of Western consumers, whereas Asians selected ‘common’ more often for the reference yogurt, as seen by the Fisher’s exact test across cultures.

**Figure 1:**
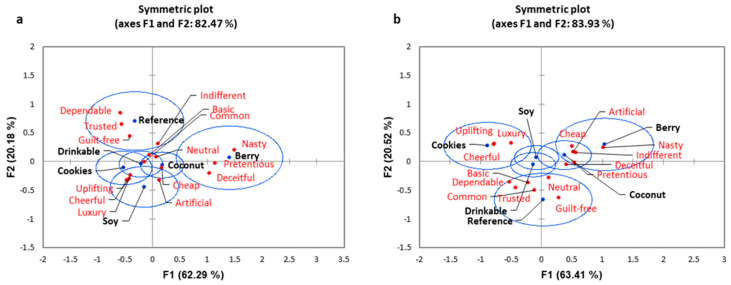
Correspondence analysis biplots for emotions comparing the two cultural groups: (**a**) Western consumers, (**b**) Asian consumers, with the confidence ellipses showing emotions related to each of the yogurt products.

A correspondence analysis using a biplot for Western consumers (Figure 1a) indicated that 82.47% of the variability observed could be attributed to the first two principal components (F1: 62.29%, F2: 20.18%). The berry was more related to the negative terms (nasty, pretentious, deceitful), whereas the reference (guilt-free, trusted, dependable) and soy (luxury, uplifting, cheerful, artificial) were more related to positive emotions. The drinkable and cookies yogurts were related to more positive and neutral terms (‘common’, ‘basic’, ‘indifferent’, ‘luxury’, ‘cheerful’, ‘uplifting’) and coconut was associated with neutral and negative responses (‘cheap’, ‘neutral’). The biplot for Asian consumers (Figure 1b) similarly indicated that 85.5% of the variability observed could be attributed to the two principal components for (F1: 63.67%, F2: 21.49%). The berry and coconut yogurts were related to more negative emotion terms (‘nasty’, ‘artificial’, ‘cheap’, ‘deceitful’); the reference dairy yogurt was related to neutral emotions (‘guilt-free’, ‘common’, ‘neutral’) and the cookies yogurt was more associated with positive emotions (‘cheerful’, ‘luxury’, ‘uplifting’). The drinkable and soy yogurts were very similar to each other.

### 3.3. Comparison of Emojis (CATA Emojis Method)

The emojis expressed by consumers of Western and Asian groups were assessed by the Cochran’s Q test (Table 3). The most selected emotion terms (*p* < 0.05) for Western consumers were: ‘smiling face 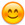
’ and ‘relieved face 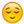
’ (reference); ‘smiling face 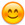
’, ‘persevering face 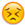
’ and ‘savoring delicious food 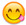
’ (coconut); ‘relieved face 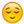
’, ‘smiling face 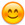
’ and ‘savoring delicious food 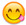
’ (drinkable); ‘relieved face 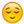
’ and ‘smiling face 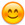
’ (soy); ‘smiling face 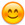
’ and ‘relieved face 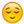
’ (cookies); ‘pensive face 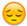
’ (berry). In comparison to Asian consumers, the top selected emotions (*p* < 0.05) for the yogurts were: ‘smiling face 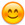
’ and ‘relieved face 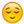
’ (reference); ‘relieved face 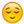
’ (coconut); ‘relieved face 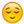
’ and ‘smiling face 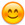
’ (drinkable); ‘smiling face 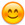
’ (soy); ‘smiling face 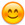
’ and ‘savoring delicious food 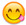
’ (cookies); ‘fearful 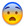
’ (berry). The reference, drinkable, soy and cookies yogurts were related to positive emojis, whereas berry was related to negative emojis, by both the cultural groups. The emojis ‘unamused 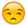
’ and ‘disappointed 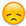
’ were similarly rated by both cultures for all the yogurt products. The berry yogurt was chosen to be more ‘pensive face 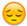
’ by Western consumers as compared to the Asians, and the cookies yogurt was related to ‘relieved face 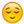
’ more often by Western consumers as compared to Asians, as seen by the Fisher’s exact test across cultures.

**Figure 2:**
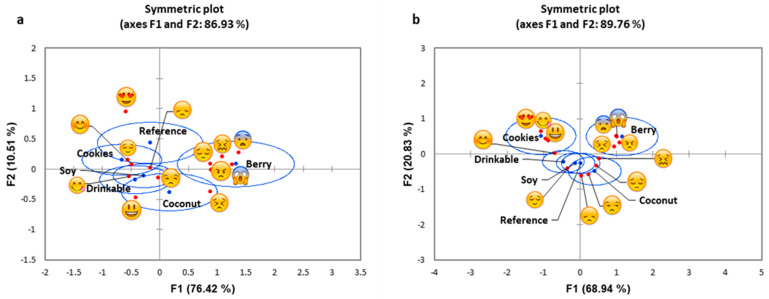
Correspondence analysis symmetric plots for emojis comparing for the two cultural groups: (**a**) Western consumers, (**b**) Asian consumers, with the confidence ellipses showing emojis related to each of the yogurt products.

The correspondence analysis biplot for Western consumers (Figure 2a) indicates that 86.93% of the variability observed could be attributed to the first two principal components (F1: 76.42%, F2: 10.51%). Meanwhile, a biplot for Asian consumers (Figure 2b) found that 89.76% variability (F1: 68.94%, F2: 20.83%) could be attributed to these components. Both yogurt products, coconut and berry, were related to more negative emojis by Asian as well as Western consumers, and the cookies was placed in the positive region by both cultural groups. In the case of Western participants, the reference, drinkable and soy yogurts were placed in between the positive and negative emotional areas, whereas Asian participants related the drinkable with positive emojis, and the reference and soy were placed in between the positive and negative regions.

### 3.4. Comparison of Facial Expression Recognition (FER) Method

Facial expressions of consumers in response to the different yogurt products were measured using the FaceReader^TM^ software. Table 4 and Table 5 show the mean values for the eight facial emotion terms, together with the valence and arousal estimates for Western and Asian consumers, respectively. No significant differences were observed among the FER attributes for Western consumers, whereas the emotions ‘surprised’ and ‘disgusted’ were significantly different (*p* < 0.05) for Asian consumers, where ‘surprised’ was rated highest for soy and lowest for berry, whereas ‘disgusted’ was rated highest for berry yogurt.

**Figure 3:**
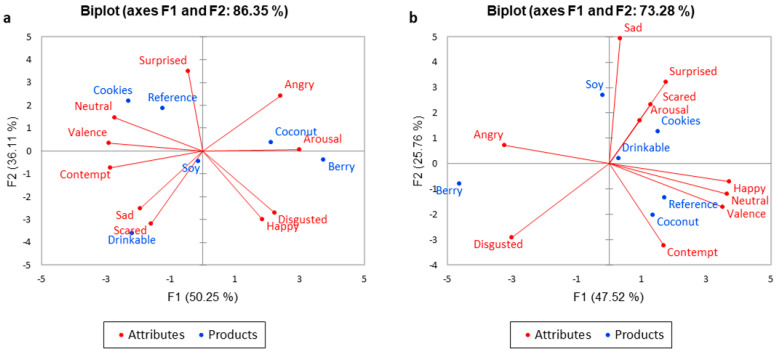
Biplots for facial expression recognition analysis comparing for the two cultural groups: (**a**) Western consumers, (**b**) Asian consumers.

The PCA biplot (Figure 3) shows that 86.35% of the variability in Western consumers’ responses could be attributed to the two principal components (F1: 50.25%, F2: 36.11%) and 73.28% of the variability could similarly be attributed to Asian consumers (F1: 47.52%, F2: 25.76%), respectively. ‘Happy’ was positively related to ‘disgusted’ and was negatively related to ‘surprised’ and ‘neutral’ in the case of Western consumers. In contrast, ‘happy’ was positively related to ‘neutral’ and ‘valence’ in the case of Asian participants and was negatively related to ‘angry’. The coconut and berry incited ‘arousal’ in the case of Western consumers and were linked to ‘angry’ emotion. The coconut was related to ‘contempt’ and berry was placed in between ‘angry’ and ‘disgusted’ for Asian consumers. The drinkable and cookies incited ‘arousal’ in the case of Asians and were linked to ‘surprised’ and ‘scared’ emotions.

### 3.5. Comparison and Relationship between Methods

The three methods—CATA emotions, CATA emojis and facial expression recognition—were examined using a linear mixed model, with overall liking as the response variable and the individual attributes as fixed effects. The percentage variance explained by each model was calculated by fitting the same explanatory variables in a linear regression model. The difference in means and standard error for each of the term in the models is shown in Table 6.

CATA emotions explained 66.0% of the variation, with ‘cheerful’, ‘neutral’, ‘trusted’, ‘indifferent’ and ‘uplifting’ being positively correlated with overall liking and showing an increase in the mean rating value of 2.29, 0.84, 1.16, 0.93 and 0.81, respectively. The terms ‘artificial’ and ‘nasty’ decreased the mean rating value by 0.87 and 2.31 units, respectively, and were negatively correlated with overall liking.

The CATA emojis accounted for 67.8% of the overall variance with the icons, with ‘heart-shaped eyes 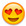
’, ‘relieved face 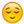
’, ‘smiling face 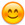
’ and ‘smiling face with mouth open 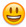
’ positively related to overall liking, showing an increase in the mean rating value of 2.08, 1.28, 1.90 and 1.35, respectively. The other emojis, including ‘angry face 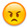
’, ‘fearful 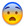
’, ‘pensive face 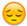
’, ‘persevering face 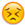
’, ‘confounded face 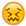
’ and ‘screaming in fear 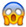
’, were negatively linked to overall liking, with a reduction in the mean rating value by 1.88, 1.69, 1.32, 1.60, 1.05 and 0.13 units, respectively. Only model 2 showed a significant effect (*p* < 0.05) of culture (Asian versus Western consumers). Western consumers had a decrease in the overall liking score of 0.36 in the model compared to Asian consumers. In other models (1 and 3), culture was not a significant (*p* > 0.05) effect.

The facial emotion recognition explained only 8.8% of the variation, with the term ‘surprised’ linked positively to overall liking, with an increase of 5.73 units in the mean value, and the term ‘disgusted’ was negatively linked to overall liking, decreasing the mean rating value by 4.73 units.

**Figure 4:**
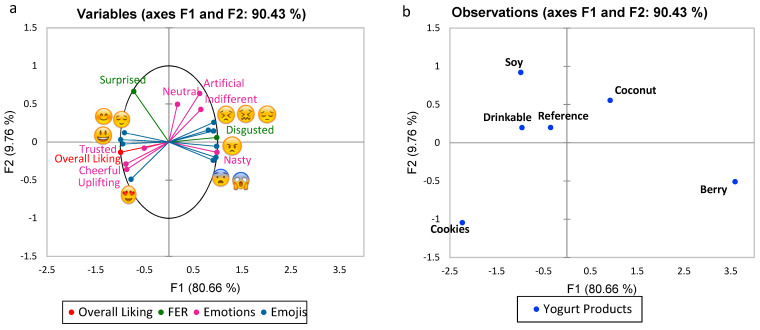
Multifactor analysis for a combination of check-all-that-apply (CATA) emotions, check-all-that-apply (CATA) emojis and facial expression recognition (FER) with overall liking ((**a**)-variables, (**b**)-yogurt products).

The multifactorial analysis (MFA) plot combined all three data sets, estimating how closely each factor was linked to other factors and the yogurt products. The MFA plot for all participants (Figure 4) explained 90.43% of the total variability (F1: 80.66%, F2: 9.76%). Overall liking was positively linked to ‘cheerful’, ‘uplifting’ and ‘trusted’ in case of the emotion terms, ‘smiling face 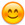
’, ‘relieved face 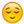
’, ‘smiling face with mouth open 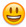
’ and ‘heart-shaped eyes 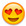
’ in case of the emojis, and ‘surprised’ in case of the biometrics. The overall liking was negatively linked to ‘artificial’, ‘nasty’, ‘indifferent’ and ‘neutral’ emotion terms, ‘fearful 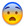
’, ‘persevering face 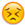
’, ‘angry face 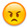
’, ‘pensive face
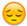
’, ‘screaming in fear 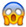
’ and ‘confounded face 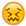
’ emojis, and ‘disgusted’ for the biometrics measurements. The soy, drinkable, reference and cookies were all positively linked to the overall liking, and the berry and coconut were linked to negative terms.

The MFA plots comparing the responses of Western and Asian participants (Supplementary Figures S1 and S2) showed that the most liked yogurt (cookies) and the least liked yogurts (berry and coconut) were similarly placed across both the cultures. Apart from this, soy and drinkable showed minor differences (although positive) across the two groups. However, the reference yogurt was related to more positive terms by Western participants and associated with slight dislike by Asians.

### 3.6. Price Perception and Purchase Intent of Yogurts

Consumers perceived the cookies yogurt to be “somewhat” to “very likely” to be purchased from a supermarket, with an average purchase intent score of 3.6 (on a scale of 5), although they considered its price to be the same as the regular yogurts that they generally consume (rated by 59.7% participants). Only the berry yogurt received the lowest score of 1.1, indicating that it was ‘not at all likely’ to be purchased. The reference, drinkable and soy yogurts were rated to be in between ‘not so likely’ and ‘somewhat likely’, although their perceived price was rated to be the ‘same’ by 41.9, 38.7 and 35.5% of participants, as compared to their regular yogurts. The data are shown in Supplementary Tables S4 and S5. The interaction for culture and yogurt products was significant for purchase intent (*p* < 0.05). Asian consumers perceived the price of cookies and drinkable yogurts to be higher. Western consumers perceived the price of the reference yogurt to be higher.

## 4. Discussion

### 4.1. Traditional Sensory Method

The advantage of using a 9-point hedonic scale is that it is simple to use by consumers and is effective in predicting the acceptance of a food product [2]. In the present study, it was successfully used to predict the liking of the six different yogurt formulations, suggesting that the cookies was the most liked and the berry was the least liked yogurt product. However, these scales could not predict any differences in the emotional responses of participants, or differences in liking across cultures, which is in contrast to some of our previous works, in which cultural differences influenced the affective responses towards different product types [29].

The hedonic scales have some drawbacks, including less freedom for the participants to express a full range of emotions [2]. Furthermore, these liking scores are limited in predicting market success. Hence, more elaborate methods, that measure consumer emotions (conscious or unconscious), need to be included for a more complete understanding of consumer food choices [30].

### 4.2. Self-Reported Responses (CATA Methods)

Apart from hedonic scale, self-reported methods of emotions or emojis has gained popularity for sensory segmentation of emotions in sensory experiments. This study shows that this approach allows differences in liking towards the same yogurt products to be better understood and is particularly useful where Western and Asian consumers use different terms to express liking towards the same yogurt type. For example, in this study, Asians selected ‘common’ to describe the reference yogurt, and the berry yogurt was associated more with the ‘pensive face 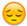
’ by Western consumers. A similar effect was found for the selection of the emotional terms in a study of chocolate tasting, as Asian and Western consumers expressed emotions relating to the consumption of chocolate differently [28]. Hence, CATA terms, when used in combination with a hedonic scale, can better explain consumer preferences and segmentation, without creating any bias [31].

Further, it has been found that emojis and emoticons can be an easy and effective way to understand food-related emotions, as consumers can relate to visual cues better than words [12]. Jaeger, Xia, Lee, Hunter, Beresford and Ares [7] found emojis (or emoticons) to be suitable for understanding the liking of a wide variety of food products by consumers. In another study by Jaeger et al. [32], emojis were successfully used to assess emotional liking towards the consumed food products (where a variety of products were served according to culture), and emojis had the potential to be used as an approach for direct measurement.

There are limitations of the self-reported methodology of emojis, as subtle differences between the samples cannot be easily detected, although this method does offer a simple and easy approach for understanding the sensory characteristics of food products [13]. Furthermore, it is important to define an emotion, so that it is easier to understand consumers’ expectations from the tasted product and also to keep a balance of positive and negative emotion terms, in order to eliminate bias [33].

### 4.3. Facial Expression Recognition Responses (FER Method)

To overcome the bias associated with self-reporting sensory terms, an unconscious method was developed that involves the recording of the facial expressions of consumers, without informing them (intrinsically). In the yogurt study, the most liked product (cookies) was related to the ‘surprised’ facial expression emotion. The least liked product (berry) was related to the ‘disgusted’ facial emotion by both cultures. ‘Happy’ was not an effective discriminator for the products based on facial expressions, as it was related to ‘disgusted’ in the case of Western participants and to ‘neutral’ for Asian participants. Previously, in a study tasting orange juices, the ‘neutral’, ‘disgusted’ and ‘angry’ facial expressions explained liking towards the tasted samples, whereas the ‘happy’ emotion was not a useful discriminator during the implicit measurement of samples. It was also shown in this implicit study that participants displayed more negative emotions, and the least liked samples were easier to differentiate [34], consistent with observations made here.

The FaceReader^TM^ emotions detected clear cultural differences here among Asian consumers, who showed a significant difference in means for ‘surprised’ and ‘disgusted’, whereas no differences were observed for Western consumers. A similar outcome was previously observed for chocolate samples, where no significant differences were observed by FaceReader^TM^ emotions, although the consumer panel was not differentiated by culture [21].

The facial expression recognition method also has some limitations. The mean value for the term ‘neutral’ was the highest for both cultures. This could be attributed to the fact that participants performed tastings in an isolated booth, which was a socially distant and neutral environment. Hence, the consumers did not show much variation in positive emotions, but negative emotions were more pronounced. In a related study, it was seen that liking was not well correlated with emotional effects but a shift in liking for the yogurts tasted was well distinguished, showing the importance of emotions experienced [35]. Negative emotions were also better displayed for disliked samples in another study of different juices [36]. This suggests that it is easier to distinguish a disliked product using facial recognition. Alvarez-Pato et al. [37] also found that facial recognition could not be used as a stand-alone method for predicting consumer acceptance of food products and this method is better applied in combination with other techniques, which is consistent with the findings here.

### 4.4. Comparison and Relationship of Methods

To compare the conscious methods of sensory analysis with the unconscious method, the overall liking scores (from hedonic scales) were used as a reference method. This comparison was performed to determine the method that best represents the variance observed and to provide the most accurate consumer insights.

The conscious methods, CATA emotions and emojis, explained more variation in the consumer data—67.8% and 66.0%, respectively. The emojis model also explained the variation for the cultural differences (*p* < 0.05), which could not be explained by the other two models. The unconscious method (FER) explained quite a low level of variation within the data (8.8%). Only two terms were relevant in the model, which were ‘surprised’ and ‘disgusted’. This outcome suggests that self-reported methods can be used alone with a hedonic scale to explain the variability in consumer response. In contrast, facial emotion recognition should be used in combination with other techniques for a better estimation of variance across consumer responses.

The MFA plot, shown in Figure 4, explains the relationship between the hedonic scales and conscious and unconscious methods of sensory analysis for the six yogurt types. The plot shows an association of positive terms with the overall liking scores and shows negative terms to be opposite to liking. In a beer tasting study [22], unconscious biometric responses were successfully integrated with conscious sensory responses for predicting consumer liking of beers. However, in another study by van Bommel et al. [38] where implicit facial expressions were compared with the explicit self-reported emotions, little overlap was found between methods, which were not directly comparable. A sensory study of organic herbal infusions [8] also found similarities between the conscious responses chosen by the consumers (EsSense Profile^®^) and unconscious responses from their facial expressions and successfully characterized emotional responses. Negative emotions were better identified by FaceReader^TM^ and positive emotions were better displayed with an EsSense Profile^®^. However, there are differing responses in the literature, as another study, involving a comparison of self-reported and implicit responses for beer, found liking scores to have a higher discrimination compared to facial expression and also found self-reported emotional responses to display the highest discrimination compared to the other two methods [39]. Another study with juices also found that a higher correlation of negative emotions from facial expression analysis was observed with hedonic liking measurements [40].

The price perception in this study for the most liked yogurt (cookies) and least liked yogurt (berries) was directly related to the liking predicted by all three sensory methods. The plain plant soy yogurt and the reference dairy yogurt were similarly liked by the participants, indicating that liking does not necessarily depend on the source of protein. However, the different food textures of the yogurts evoked different emotions in our study. Reports have shown that emotions are more linked to the intrinsic properties of a food product and can vary accordingly, even if the liking scores are the same. The relation between the emotions and liking is not straightforward [41], also confirmed in our study with yogurts. Further, familiarity or neophobia is an important parameter that plays an significant role in the representation of emotions across cultures [42]. In a study with Greek yogurts, different textures produced varying consumer responses and liking [43]. Hence, it is important to understand the emotions linked to the tasted yogurts, as in our case, to understand food choice. Studies have shown that different cultures understand the same set of emotions, expressing these differently [33]. Other structural and compositional factors may also affect liking scores, which were not explored in detail in this study.

## 5. Conclusions

The traditional sensory methods using a hedonic scale offer benefits in terms of their speed and ease of use but have shortcomings in quantifying the full range of emotional responses and reasons behind liking or disliking of yogurt products. Conscious and unconscious methods can also be combined with a hedonic scale for a better understanding of consumer perceptions. These advanced approaches were found to be useful in measuring the cultural differences in liking towards yogurt products, even though the hedonic scale liking scores were similar. The conscious approach using the CATA methodology explained a higher proportion of variance for yogurt-tasting data. In contrast, the novel facial expression recognition approach distinguished the acceptability of different yogurt samples—more specifically, disliked samples—even though this method explained a very low proportion of the variability in the consumer data. The effect of culture was shown by the CATA and FER methods, where each culture displayed a different set of emotions for the tasted yogurts. Although an effect of culture was not shown by the traditional hedonic scale method, this effect was seen on the price perception of the tasted yogurts. At least in the evaluation of yogurt formulations, it is therefore recommended that these different methods be combined for a more comprehensive explanation of consumer perceptions and expectations in a multi-cultural environment.

## 6. Limitations and Future Research

The limited sample size in the experiment may present certain constraints in interpreting the results. Therefore, future research is recommended by increasing the sample size of participants. Additionally, the dependence of gender can be studied through all three methods tested in the experiment once the sample size is increased. Furthermore, the sensitivity of tasting can be checked for a second and third tasting trial using the FER method (that is, having multiple tastings during the sensory session) to understand how the result could vary with more than one trial, for the same participant. The FER can also be tested in different locations for a more ecologically valid experience, rather than a closed isolated setting, which might help in generating fewer facial expressions. Product parameters, such as particle size and emulsifiers, are also important factors contributing to the liking of yogurts, and these parameters can be measured and linked to the emotions to improve our understanding of liking in further studies.

## Figures and Tables

**Table 1:** Comparison of overall liking scores of the different yogurt products by Asian and Western consumers.

Yogurt Product Type	Product Code (as Used in This Study)	Overall Liking Scores (Asian Consumers)	Overall Liking Scores (Western Consumers)
Dairy Greek yogurt	Reference	5.03 ± 2.33 ^cd^	5.39 ± 2.26 ^bc^
(plain)			
Coconut-based yogurt (plain)	Coconut	3.73 ± 2.05 ^d^	4.39 ± 2.30 ^c^
Drinkable yogurt (sweetened)	Drinkable	6.15 ± 2.16 ^b^	5.51 ± 2.11 ^b^
Soy-based yogurt	Soy	5.45 ± 2.25 ^bc^	5.64 ± 2.11 ^b^
(plain)			
Dairy yogurt with crunchies (sweetened)	Cookies	7.82 ± 0.94 ^a^	6.87 ± 1.43 ^a^
Dairy yogurt with berries (sweetened)	Berry	1.90 ± 0.92 ^e^	2.02 ± 1.12 ^d^
* F-value		38.93	20.86

^a,b,c,d,e^ Means with different superscripts in each column indicate significant differences (*p* < 0.05) by Fisher’s least square difference test. Highest value with ‘a’ in superscript. The data are presented as mean ± standard deviation. * as an indicator of significance.

**Table 2:** Cochran’s Q test for emotion terms shown for the two cultural groups (Western consumers, Asian consumers) comparing terms within the culture, and using Fisher’s test to compare terms across cultures.

		Western Consumers								Asian Consumers					
Attributes	Reference	Coconut	Drinkable	Soy	Cookies	Berry	*p*-Values	Attributes	Reference	Coconut	Drinkable	Soy	Cookies	Berry	*p*-Values
Cheerful **	0.17 ^bcd^	0.14 ^cd^	0.38 ^abc^	0.52 ^ab^	0.59 ^a^	0.00 ^d^	<0.001	Cheerful **	0.12 ^xy^	0.09 ^xy^	0.36 ^y^	0.39 ^y^	0.88 ^z^	0.00 ^x^	<0.001
Neutral ^ns^	0.21 ^a^	0.24 ^a^	0.35 ^a^	0.17 ^a^	0.14 ^a^	0.17 ^a^	0.280	Neutral ^ns^	0.24 ^x^	0.21 ^x^	0.18 ^x^	0.12 ^x^	0.09 ^x^	0.09 ^x^	0.341
Nasty **	0.10 ^b^	0.21 ^b^	0.03 ^b^	0.07 ^b^	0.00 ^b^	0.72 ^a^	<0.001	Nasty **	0.15 ^xy^	0.21 ^xy^	0.09 ^x^	0.09 ^x^	0.00 ^x^	0.55 ^y^	<0.001
Luxury *	0.07 ^b^	0.24 ^ab^	0.10 ^b^	0.14 ^ab^	0.38 ^a^	0.00 ^b^	0.001	Luxury **	0.06 ^x^	0.21 ^xy^	0.03 ^x^	0.18 ^xy^	0.33 ^y^	0.00 ^x^	0.000
Guilt-free ^ns^	0.24 ^a^	0.14 ^a^	0.10 ^a^	0.07 ^a^	0.17 ^a^	0.00 ^a^	0.077	Guilt-free *	0.24 ^x^	0.09 ^x^	0.09 ^x^	0.03 ^x^	0.03 ^x^	0.09 ^x^	0.045
Deceitful *	0.00 ^b^	0.10 ^ab^	0.07 ^ab^	0.10 ^ab^	0.03 ^b^	0.28 ^a^	0.003	Deceitful ^ns^	0.09 ^x^	0.09 ^x^	0.12 ^x^	0.15 ^x^	0.00 ^x^	0.12 ^x^	0.313
Trusted **	0.34 ^a^	0.03 ^b^	0.14 ^ab^	0.07 ^b^	0.24 ^ab^	0.00 ^b^	0.000	Trusted *	0.24 ^y^	0.03 ^xy^	0.09 ^xy^	0.15 ^xy^	0.18 ^xy^	0.00 ^x^	0.007
Basic ^ns^	0.24 ^a^	0.24 ^a^	0.31 ^a^	0.21 ^a^	0.10 ^a^	0.10 ^a^	0.193	Basic *	0.36 ^y^	0.18 ^xy^	0.33 ^y^	0.15 ^xy^	0.24 ^xy^	0.03 ^x^	0.008
Pretentious ^ns^	0.00 ^a^	0.10 ^a^	0.00 ^a^	0.00 ^a^	0.00 ^a^	0.07 ^a^	0.060	Pretentious ^ns^	0.06 ^x^	0.12 ^x^	0.00 ^x^	0.09 ^x^	0.00 ^x^	0.06 ^x^	0.094
Uplifting **	0.10 ^bc^	0.20 ^abc^	0.28 ^abc^	0.31 ^ab^	0.45 ^a^	0.00 ^c^	0.000	Uplifting **	0.06 ^x^	0.09 ^x^	0.24 ^xy^	0.15 ^x^	0.51 ^y^	0.00 ^x^	<0.001
Indifferent ^ns^	0.14 ^a^	0.21 ^a^	**0.14 ^a^**	0.03 ^a^	0.03 ^a^	0.07 ^a^	0.210	Indifferent ^ns^	0.06 ^x^	0.09 ^x^	**0.00 ^x^**	0.09 ^x^	0.03 ^x^	0.12 ^x^	0.315
Cheap ^ns^	0.07 ^a^	0.03 ^a^	0.17 ^a^	0.14 ^a^	0.10 ^a^	0.14 ^a^	0.562	Cheap *	0.06 ^x^	0.21 ^x^	0.27 ^x^	0.18 ^x^	0.06 ^x^	0.33 ^x^	0.015
Dependable *	0.24 ^a^	0.03 ^ab^	0.07 ^ab^	0.00 ^b^	0.17 ^ab^	0.00 ^b^	0.002	Dependable ^ns^	0.09 ^x^	0.00 ^x^	0.06 ^x^	0.06 ^x^	0.09 ^x^	0.00 ^x^	0.247
Artificial *	0.10 ^b^	0.38 ^ab^	0.24 ^ab^	0.48 ^a^	0.24 ^ab^	0.24 ^ab^	0.023	Artificial **	0.18 ^xy^	0.52 ^z^	0.27 ^xyz^	0.36 ^xyz^	0.06 ^x^	0.46 ^yz^	0.000
Common ^ns^	**0.07 ^a^**	0.10 ^a^	0.10 ^a^	0.07 ^a^	0.10 ^a^	0.03 ^a^	0.911	Common *	**0.36 ^y^**	0.09 ^x^	0.15 ^xy^	0.21 ^xy^	0.15 ^xy^	0.06 ^x^	0.013

** Indicates significant differences between samples according to Cochran’s Q test at *p* < 0.001. * Indicates significant differences between samples according to Cochran’s Q test at *p* < 0.05. ^ns^ Indicates no significant difference between samples according to Cochran’s Q test at *p* > 0.05, with ‘a’ the largest in case of Western and ‘x’ in case of Asian consumers. Significant differences between pairs of samples across cultures according to Fisher’s exact test are indicated by bold terms. Fisher’s exact test is generally more conservative than Cochran’s Q test, so the paired comparisons may all be not significant even if the overall test is significant.

**Table 3:** Cochran’s Q test for emoji terms shown for the two cultural groups (Western consumers, Asian consumers) comparing terms within the culture, and using Fisher’s test to compare terms across cultures.

	Western Consumers								Asian Consumers						
Attributes	Reference	Coconut	Drinkable	Soy	Cookies	Berry	*p*-Values	Attributes	Reference	Coconut	Drinkable	Soy	Cookies	Berry	*p*-Values
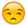 ^ns^	0.14 ^a^	0.21 ^a^	0.21 ^a^	0.10^a^	**0.14^a^**	0.14 ^a^	0.817	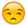 ^ns^	0.12 ^xy^	0.24 ^x^	0.12 ^xy^	0.15 ^xy^	**0.00 ^y^**	0.09 ^xy^	0.087
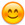 *	0.28 ^ab^	0.21 ^ab^	0.24 ^ab^	0.31 ^ab^	0.41 ^a^	0.00 ^b^	0.007	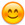 **	0.15 ^yz^	0.09 ^z^	0.42 ^xy^	0.30 ^xyz^	0.52 ^x^	0.00 ^z^	<0.001
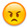 ^ns^	0.03 ^a^	0.03 ^a^	0.03 ^a^	0.00 ^a^	0.00 ^a^	0.10 ^a^	0.267	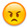 *	0.00 ^y^	0.09 ^xy^	0.00 ^y^	0.03 ^xy^	0.00 ^y^	0.18 ^x^	0.001
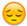 *	0.07 ^b^	0.14 ^ab^	0.03 ^b^	0.07 ^b^	0.03 ^b^	**0.35 ^a^**	0.001	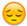 ^ns^	0.06 ^x^	0.15 ^x^	0.06 ^x^	0.09 ^x^	0.00 ^x^	**0.12 ^x^**	0.255
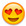 ^ns^	0.10 ^a^	0.00 ^a^	0.00 ^a^	0.03 ^a^	0.10 ^a^	0.00 ^a^	0.098	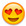 *	0.03 ^xy^	0.00 ^y^	0.03 ^xy^	0.03 ^xy^	0.18 ^x^	0.00 ^y^	0.004
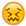 **	0.10 ^ab^	0.10 ^ab^	0.00 ^b^	0.03 ^b^	0.00 ^b^	0.31 ^a^	0.000	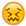 *	0.21 ^x^	0.09 ^x^	0.03 ^x^	0.12 ^x^	0.00 ^x^	0.21 ^x^	0.026
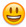 ^ns^	0.03 ^a^	**0.21 ^a^**	0.21 ^a^	0.17 ^a^	0.17 ^a^	0.00 ^a^	0.051	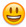 ^*^	0.09 ^xy^	**0.00 ^y^**	0.06 ^y^	0.09 ^xy^	0.27 ^x^	0.00 ^y^	0.001
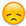 ^ns^	0.28 ^a^	0.14 ^a^	0.24 ^a^	0.35 ^a^	0.07 ^a^	0.10 ^a^	0.050	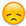 ^ns^	0.15 ^x^	0.18 ^x^	0.09 ^x^	0.18 ^x^	0.03 ^x^	0.03 ^x^	0.146
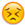 ^**^	0.00 ^b^	0.17 ^ab^	0.03 ^b^	0.14 ^ab^	0.00 ^b^	0.31 ^a^	0.000	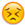 **	0.06 ^y^	0.15 ^xy^	0.00 ^y^	0.09 ^y^	0.00 ^y^	0.33 ^x^	<0.001
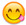 *	0.10 ^ab^	0.17 ^ab^	0.24 ^ab^	0.21 ^ab^	0.35 ^a^	0.00 ^b^	0.005	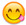 **	0.06 ^y^	0.03 ^y^	0.15 ^y^	0.09 ^xy^	0.42 ^x^	0.00 ^y^	<0.001
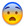 **	0.07 ^b^	0.07 ^b^	0.00 ^b^	0.00 ^b^	0.00 ^b^	0.31 ^a^	<0.001	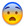 **	0.06 ^y^	0.09 ^y^	0.06 ^y^	0.06 ^y^	0.00 ^y^	0.49 ^x^	<0.001
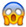 *	0.00	0.00	0.03 ^ab^	0.03 ^ab^	0.00	0.17 ^b^	0.004	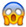 *	0.06 ^xy^	0.03 ^y^	0.03 ^y^	0.00 ^y^	0.00 ^y^	0.21 ^x^	0.001
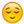 **	0.28 ^ab^	0.07 ^b^	0.45 ^a^	0.35 ^ab^	**0.55 ^a^**	0.03 ^b^	<0.001	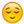 *	0.18 ^xy^	0.18 ^xy^	0.30 ^x^	0.12 ^xy^	**0.15 ^xy^**	0.00 ^y^	0.014

** Indicates significant differences between samples according to Cochran’s Q test at *p* < 0.001. * Indicates significant differences between samples according to Cochran’s Q test at *p* < 0.05. ^ns^ Indicates no significant difference between samples according to Cochran’s Q test at *p* > 0.05, with ‘a’ the largest in case of Western and ‘x’ in case of Asian consumers. Significant differences between pairs of samples according to Fisher’s exact test are indicated by bold terms. Fisher’s exact test is generally more conservative than Cochran’s Q test, so the paired comparisons may all be not significant even if the overall test is significant.

**Table 4:** Mean values for the biometric emotions for Western consumers.

Product Code	Neutral ^NS^	Happy ^NS^	Sad ^NS^	Angry ^NS^	Surprised ^NS^	Scared ^NS^	Disgusted ^NS^	Contempt ^NS^	Valence ^NS^	Arousal ^NS^
Reference	0.53 ± 0.21	0.02 ± 0.02	0.12 ± 0.18	0.12 ± 0.14	0.04 ± 0.05	0.02 ± 0.02	0.07 ± 0.11	0.01 ± 0.02	−0.23 ± 0.19	0.30 ± 0.11
Coconut	0.51 ± 0.20	0.03 ± 0.08	0.10 ± 0.14	0.14 ± 0.22	0.03 ± 0.04	0.02 ± 0.02	0.11 ± 0.13	0.01 ± 0.02	−0.24 ± 0.25	0.32 ± 0.12
Drinkable	0.51 ± 0.17	0.03 ± 0.10	0.16 ± 0.18	0.08 ± 0.10	0.02 ± 0.03	0.03 ± 0.04	0.11 ± 0.16	0.02 ± 0.03	−0.22 ± 0.23	0.27 ± 0.12
Soy	0.54 ± 0.21	0.03 ± 0.05	0.15 ± 0.19	0.11 ± 0.13	0.03 ± 0.04	0.02 ± 0.02	0.10 ± 0.13	0.01 ± 0.01	−0.25 ± 0.20	0.28 ± 0.11
Cookies	0.55 ± 0.18	0.01 ± 0.02	0.12 ± 0.15	0.13 ± 0.13	0.03 ± 0.05	0.02 ± 0.02	0.07 ± 0.08	0.01 ± 0.02	−0.22 ± 0.15	0.26 ± 0.12
Berry	0.47 ± 0.19	0.03 ± 0.04	0.11 ± 0.17	0.16 ± 0.18	0.03 ± 0.04	0.02 ± 0.02	0.12 ± 0.12	0.01 ± 0.02	−0.27 ± 0.20	0.33 ± 0.11
F-value	0.65	0.53	0.51	0.93	0.39	0.69	0.97	0.93	0.20	1.71

^NS^ indicates no significant differences in that column (*p* > 0.05) by the Fisher’s least square difference test. The data are presented as mean ± standard deviation.

**Table 5:** Mean values for the biometric emotions for Asian consumers.

Product Code	Neutral ^NS^	Happy ^NS^	Sad ^NS^	Angry ^NS^	Surprised	Scared ^NS^	Disgusted	Contempt ^NS^	Valence ^NS^	Arousal ^NS^
Reference	0.41 ± 0.14	0.05 ± 0.05	0.20 ± 0.17	0.10 ± 0.08	0.06 ± 0.10 ^ab^	0.04 ± 0.04	0.09 ± 0.09 ^b^	0.01 ± 0.02	−0.22 ± 0.17	0.30 ± 0.14
Coconut	0.41 ± 0.11	0.05 ± 0.05	0.19 ± 0.14	0.11 ± 0.12	0.06 ± 0.08 ^ab^	0.05 ± 0.04	0.08 ± 0.07 ^b^	0.01 ± 0.02	−0.22 ± 0.15	0.30 ± 0.12
Drinkable	0.40 ± 0.12	0.04 ± 0.05	0.23 ± 0.18	0.11 ± 0.14	0.08 ± 0.12 ^ab^	0.03 ± 0.03	0.06 ± 0.08 ^b^	0.01 ± 0.01	−0.26 ± 0.21	0.29 ± 0.10
Soy	0.38 ± 0.13	0.04 ± 0.06	0.25 ± 0.19	0.12 ± 0.13	0.09 ± 0.13 ^a^	0.05 ± 0.06	0.05 ± 0.05 ^b^	0.01 ± 0.01	−0.27 ± 0.19	0.29 ± 0.14
Cookies	0.40 ± 0.15	0.05 ± 0.06	0.23 ± 0.20	0.11 ± 0.12	0.05 ± 0.07 ^ab^	0.05 ± 0.04	0.04 ± 0.05 ^b^	0.01 ± 0.04	−0.23 ± 0.22	0.82 ± 2.84
Berry	0.36 ± 0.11	0.03 ± 0.04	0.20 ± 0.16	0.12 ± 0.13	0.03 ± 0.04 ^b^	0.04 ± 0.05	0.15 ± 0.17 ^a^	0.01 ± 0.01	−0.31 ± 0.15	0.33 ± 0.17
F-value	0.69	0.68	0.59	0.16	1.34	0.65	5.26	0.11	1.07	1.03

^a,b^ Means with different superscripts in each column indicate significant differences (*p* < 0.05) by the Fisher’s least square difference test. ^NS^ indicates no significant differences in that column (*p* > 0.05). Highest value indicated with ‘a’ in superscript. The data are presented as mean ± standard deviation.

**Table 6:** A linear mixed model for each of the methods, taking ‘overall liking’ as the response variable and representing percentage variance explained by each method.

Model	Fixed Factor	Difference between Means	Standard Error (SE)	*p* Value	F-Value	Random Factor	Overall Mean	Standard Error of Difference	Percentage Variance
Check-all-that-apply (CATA) emotions	Artificial	−0.87	0.18	<0.001	24.16	Yogurt Samples	4.91	0.20	66
	Cheerful	2.29	0.21	<0.001	116.49				
	Nasty	−2.31	0.22	<0.001	106.11				
	Neutral	0.84	0.21	<0.001	15.86				
	Trusted	1.16	0.25	<0.001	21.77				
	Uplifting	0.81	0.23	<0.001	11.17				
	Indifferent	0.93	0.28	0.001	10.67				
Check-all-that-apply (CATA) emojis	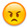	−1.88	0.38	<0.001	24.59	None	4.91	0.14	67.8
	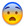	1.69	0.27	<0.001	38.15				
	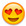	2.08	0.38	<0.001	30.58				
	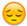	−1.32	0.27	<0.001	23.86				
	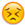	−1.60	0.25	<0.001	39.28				
	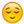	1.28	0.19	<0.001	43.97				
	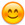	1.90	0.19	<0.001	101.36				
	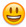	1.35	0.25	<0.001	29.30				
	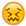	−1.05	0.26	<0.001	16.28				
	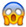	−0.13	0.37	<0.001	12.49				
	Culture	−0.36	0.15	0.019	5.52				
FER	Disgusted	−4.73	1.15	<0.001	11.11	Yogurt Samples	5.52	0.29	8.8
	Surprised	5.73	1.72	<0.001	16.94				

## Supplementary Materials

The following are available online at https://www.mdpi.com/article/10.3390/foods10061237/s1. Table S1: List of check-all-that-apply (CATA) emotion terms given to the consumers. Table S2: List of check-all-that-apply (CATA) emojis given to the consumers. Table S3: List of emotions (unconscious responses) represented by FaceReader^TM^. Table S4: Purchase intent of yogurts as rated by participants. Table S5: Price perception of yogurts as rated by participants in reference to regular yogurts (all values are in percentages).

## References

[1] Iannario M., Manisera M., Piccolo D., Zuccolotto P. (2012). Sensory analysis in the food industry as a tool for marketing decisions. Adv. Data Anal. Classif..

[2] Lim J. (2011). Hedonic scaling: A review of methods and theory. Food Qual. Prefer..

[3] Prescott J., Bell G. (1995). Cross-cultural determinants of food acceptability: Recent research on sensory perceptions and preferences. Trends Food Sci. Technol..

[4] He W., Boesveldt S., de Graaf C., de Wijk R.A. (2016). The relation between continuous and discrete emotional responses to food odors with facial expressions and non-verbal reports. Food Qual. Prefer..

[5] Ng M., Chaya C., Hort J. (2013). Beyond liking: Comparing the measurement of emotional response using EsSense Profile and consumer defined check-all-that-apply methodologies. Food Qual. Prefer..

[6] Meiselman H.L. (2013). The future in sensory/consumer research: Evolving to a better science. Food Qual. Prefer..

[7] Jaeger S.R., Xia Y., Lee P., Hunter D.C., Beresford M.K., Ares G. (2018). Emoji questionnaires can be used with a range of population segments: Findings relating to age, gender and frequency of emoji/emoticon use. Food Qual. Prefer..

[8] Rocha C., Lima R., Moura A., Costa T., Cunha L. (2019). Implicit evaluation of the emotional response to premium organic herbal infusions through a temporal dominance approach: Development of the temporal dominance of facial emotions (TDFE). Food Qual. Prefer..

[9] Ares G., Barreiro C., Deliza R., Giménez A., Gámbaro A. (2010). Application of a check-all-that-apply question to the development of chocolate milk desserts. J. Sens. Stud..

[10] Giacalone D., Bredie W., Frøst M.B. (2013). “All-In-One Test” (AI1): A rapid and easily applicable approach to consumer product testing. Food Qual. Prefer..

[11] Son J.-S., Do V.B., Kim K.-O., Cho M.S., Suwonsichon T., Valentin D. (2014). Understanding the effect of culture on food representations using word associations: The case of “rice” and “good rice”. Food Qual. Prefer..

[12] Vidal L., Ares G., Jaeger S. (2016). Use of emoticon and emoji in tweets for food-related emotional expression. Food Qual. Prefer..

[13] Varela P., Ares G. (2012). Sensory profiling, the blurred line between sensory and consumer science. A review of novel methods for product characterization. Food Res. Int..

[14] De Wijk R.A., He W., Mensink M.G.J., Verhoeven R.H.G., De Graaf C. (2014). ANS Responses and Facial Expressions Differentiate between the Taste of Commercial Breakfast Drinks. PLoS ONE.

[15] Masupha L., Zuva T., Ngwira S., Esan O. Face recognition techniques, their advantages, disadvantages and performance evaluation. Proceedings of the 2015 International Conference on Computing, Communication and Security (ICCCS).

[16] Bartkiene E., Steibliene V., Adomaitiene V., Juodeikiene G., Cernauskas D., Lele V., Klupsaite D., Zadeike D., Jarutiene L., Guiné R.P.F. (2019). Factors Affecting Consumer Food Preferences: Food Taste and Depression-Based Evoked Emotional Expressions with the Use of Face Reading Technology. BioMed Res. Int..

[17] Terzis V., Moridis C.N., Economides A.A. (2011). Measuring instant emotions based on facial expressions during computer-based assessment. Pers. Ubiquitous Comput..

[18] Jain A., Ross A., Prabhakar S. (2004). An Introduction to Biometric Recognition. IEEE Trans. Circuits Syst. Video Technol..

[19] Liu J., Bech A.C., Waehrens S.S., Bredie W.L. (2021). Perception and liking of yogurts with different degrees of granularity in relation to ethnicity, preferred oral processing and lingual tactile acuity. Food Qual. Prefer..

[20] Viejo C.G., Zhang H., Khamly A., Xing Y., Fuentes S. (2021). Coffee Label Assessment Using Sensory and Biometric Analysis of Self-Isolating Panelists through Videoconference. Beverages.

[21] Gunaratne T.M., Fuentes S., Gunaratne N.M., Torrico D.D., Viejo C.G., Dunshea F.R. (2019). Physiological Responses to Basic Tastes for Sensory Evaluation of Chocolate Using Biometric Techniques. Foods.

[22] Viejo C.G., Fuentes S., Howell K., Torrico D.D., Dunshea F.R. (2019). Integration of non-invasive biometrics with sensory analysis techniques to assess acceptability of beer by consumers. Physiol. Behav..

[23] Leitch K., Duncan S., O’Keefe S., Rudd R., Gallagher D. (2015). Characterizing consumer emotional response to sweeteners using an emotion terminology questionnaire and facial expression analysis. Food Res. Int..

[24] McCarthy K., Parker M., Ameerally A., Drake S., Drake M. (2017). Drivers of choice for fluid milk versus plant-based alternatives: What are consumer perceptions of fluid milk?. J. Dairy Sci..

[25] Jeske S., Zannini E., Arendt E.K. (2018). Past, present and future: The strength of plant-based dairy substitutes based on gluten-free raw materials. Food Res. Int..

[26] Fuentes S., Viejo C.G., Torrico D.D., Dunshea F.R. (2018). Development of a Biosensory Computer Application to Assess Physiological and Emotional Responses from Sensory Panelists. Sensors.

[27] Gelici-Zeko M., Lutters D., Klooster R.T., Weijzen P.L.G. (2012). Studying the Influence of Packaging Design on Consumer Perceptions (of Dairy Products) Using Categorizing and Perceptual Mapping. Packag. Technol. Sci..

[28] Gunaratne T.M., Viejo C.G., Fuentes S., Torrico D.D., Gunaratne N.M., Ashman H., Dunshea F.R. (2019). Development of emotion lexicons to describe chocolate using the Check-All-That-Apply (CATA) methodology across Asian and Western groups. Food Res. Int..

[29] Torrico D.D., Fuentes S., Viejo C.G., Ashman H., Dunshea F.R. (2019). Cross-cultural effects of food product familiarity on sensory acceptability and non-invasive physiological responses of consumers. Food Res. Int..

[30] Gutjar S., De Graaf C., Kooijman V., De Wijk R.A., Nys A., Ter Horst G.J., Jager G. (2015). The role of emotions in food choice and liking. Food Res. Int..

[31] Ares G., Jaeger S. (2015). Examination of sensory product characterization bias when check-all-that-apply (CATA) questions are used concurrently with hedonic assessments. Food Qual. Prefer..

[32] Jaeger S., Vidal L., Kam K., Ares G. (2017). Can emoji be used as a direct method to measure emotional associations to food names? Preliminary investigations with consumers in USA and China. Food Qual. Prefer..

[33] Meiselman H.L. (2015). A review of the current state of emotion research in product development. Food Res. Int..

[34] Danner L., Sidorkina L., Joechl M., Duerrschmid K. (2014). Make a face! Implicit and explicit measurement of facial expressions elicited by orange juices using face reading technology. Food Qual. Prefer..

[35] Mojet J., Dürrschmid K., Danner L., Jöchl M., Heiniö R.-L., Holthuysen N., Köster E. (2015). Are implicit emotion measurements evoked by food unrelated to liking?. Food Res. Int..

[36] Danner L., Haindl S., Joechl M., Duerrschmid K. (2014). Facial expressions and autonomous nervous system responses elicited by tasting different juices. Food Res. Int..

[37] Álvarez-Pato V.M., Sánchez C.N., Domínguez-Soberanes J., Méndoza-Pérez D.E., Velázquez R. (2020). A Multisensor Data Fusion Approach for Predicting Consumer Acceptance of Food Products. Foods.

[38] van Bommel R., Stieger M., Visalli M., de Wijk R., Jager G. (2020). Does the face show what the mind tells? A comparison between dynamic emotions obtained from facial expressions and Temporal Dominance of Emotions (TDE). Food Qual. Prefer..

[39] Beyts C., Chaya C., Dehrmann F., James S., Smart K., Hort J. (2017). A comparison of self-reported emotional and implicit responses to aromas in beer. Food Qual. Prefer..

[40] Zhi R., Wan J., Zhang D., Li W. (2018). Correlation between hedonic liking and facial expression measurement using dynamic affective response representation. Food Res. Int..

[41] King S.C., Meiselman H.L. (2010). Development of a method to measure consumer emotions associated with foods. Food Qual. Prefer..

[42] Barrena R., Sánchez M. (2013). Neophobia, personal consumer values and novel food acceptance. Food Qual. Prefer..

[43] Desai N., Shepard L., Drake M. (2013). Sensory properties and drivers of liking for Greek yogurts. J. Dairy Sci..

