# Supplementary material for: Differences in Hedonic Responses, Facial Expressions and Self-Reported Emotions of Consumers Using Commercial Yogurts: A Cross-Cultural Study
Source: Foods. 2021 May 29;10(6):1237. doi: 10.3390/foods10061237 (PMC8227163; doi:10.3390/foods10061237)
Supplement: Supplementary file 1 [file foods-10-01237-s001.zip › foods-1200284-supplementary.pdf]

Supplementary Data

Table S1. List of check-all-that-apply (CATA) emotion terms given to the consumers.

Positive	Neutral	Negative
Cheerful	Neutral	Nasty
Luxury	Guilt-free	Deceitful
Trusted	Basic	Pretentious
Uplifting	Indifferent	Cheap
Dependable	Common	Artificial

Table S2. List of check-all-that-apply (CATA) emojis given to the consumers.

Classification	Emoticon	Meaning (or description)
Positive	😊	smiling face
	😋	savoring delicious food
	😌	relieved face
	😍	heart-shaped eyes
	😃	smiling face with mouth open
Negative	😞	disappointed
	😣	persevering face
	😠	angry face
	😔	pensive face
	😖	confounded face
	😱	screaming in fear
	😨	fearful
Unclassified	😒	unamused

Table S3. List of emotions (unconscious responses) represented by FaceReader™

Neutral	Happy	Sad	Angry
Contempt	Surprised	Disgusted	Scared

Table S4. Purchase Intent of yogurts as rated by participants

Product code	Mean Score
Reference	2.60 ± 1.25^{b}
Coconut	1.97 ± 1.17^{c}
Drinkable	2.76 ± 1.22^{b}
Soy	2.52 ± 1.26^{b}
Cookies	3.63 ± 1.04^{a}
Berry	1.10 ± 0.30^{d}

$^{a, b, c, d}$ Means with different superscripts in each column indicate significant differences ($p < 0.05$) by the Fischer's LSD test. Highest value with 'a' in super-script. The rating scales from 1–5 is mentioned below:

Not at all likely	Not so likely	Somewhat likely	Very likely	Extremely likely
1	2	3	4	5

Table S5. Price Perception of yogurts as rated by participants in reference to their regular yogurts (all values are in percentages)

Product code	Wouldn't buy	Less	Same	More
Reference	32.3	22.6	**41.9**	3.2
Coconut	**58.1**	12.9	24.2	4.8
Drinkable	24.2	33.9	**38.7**	3.2
Soy	30.6	22.6	**35.5**	11.3
Cookies	4.8	17.7	**59.7**	17.7
Berry	**91.9**	6.5	1.6	0.0

The rating scales explanation is mentioned below:

Price Perception Label	Price Perception by Consumers
Wouldn't buy	Wouldn't want to buy when compared to their regular yogurts
Less	Would pay less as compared to their regular yogurts
Same	Would pay the same value as compared to their regular yogurts
More	Willing to pay more as compared to their regular yogurts

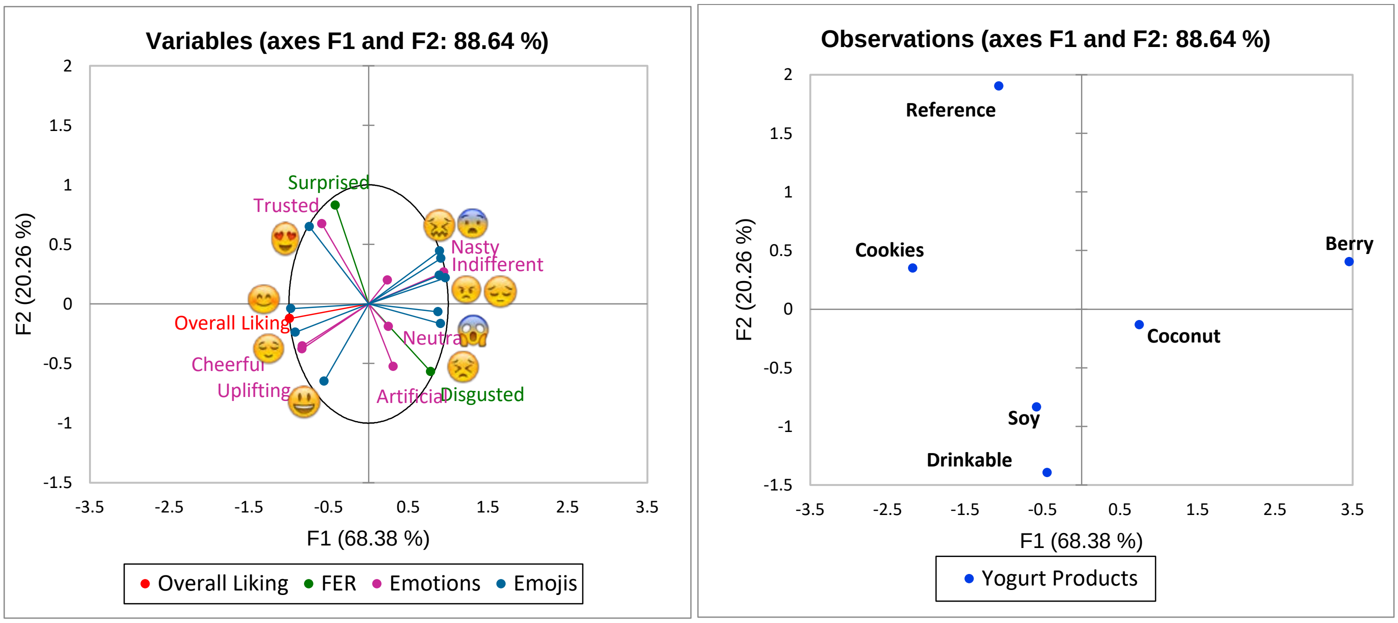

Figure S1. Multifactor analysis for a combination of CATA – emotions, CATA – emojis and FER with overall liking (a – variables, b – yogurt products) for Westerns participants.

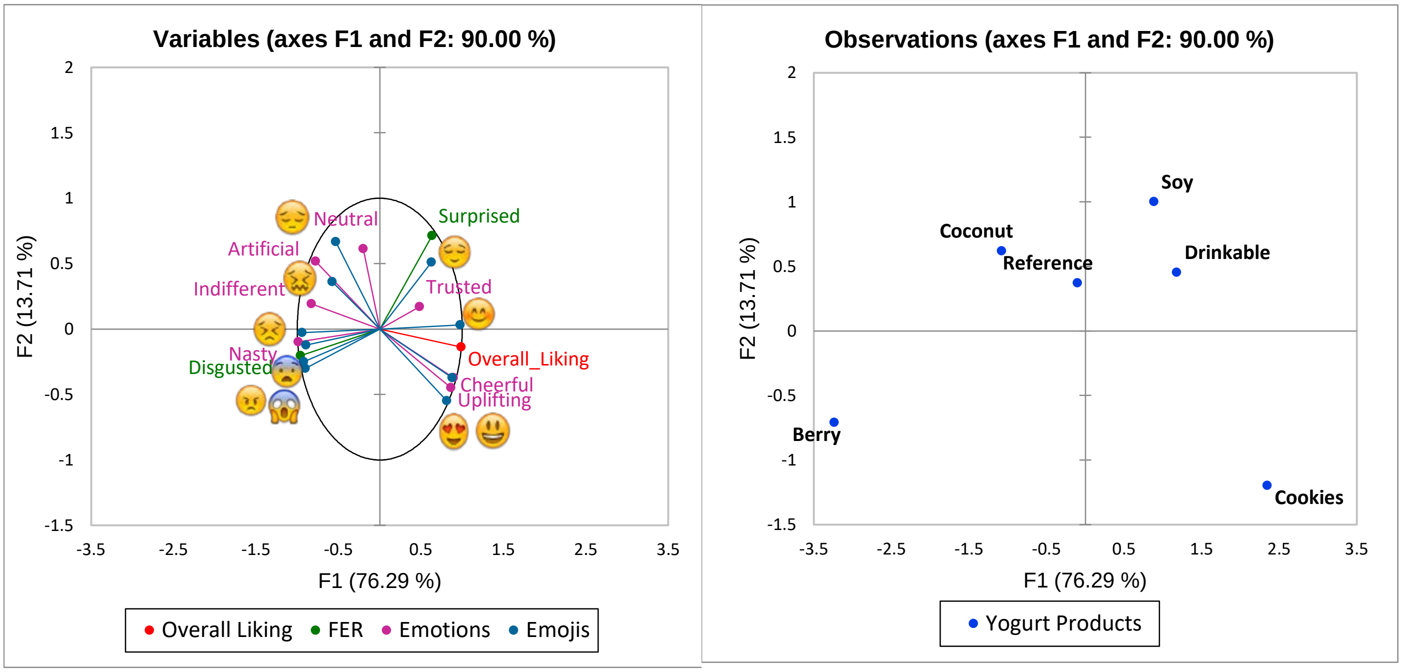

Figure S2. Multifactor analysis for a combination of CATA – emotions, CATA – emojis and FER with overall liking (a – variables, b – yogurt products) for Asian participants
